# Sex-Based Differences in Dissection Patterns and Surgical Strategies in Tear-Oriented Repair for Acute Type A Aortic Dissection

**DOI:** 10.5761/atcs.lte.26-00124

**Published:** 2026-08-07

**Authors:** Izatullah Jalalzai

**Affiliations:** Department of Cardiovascular Surgery, Ataturk University Faculty of Medicine, Erzurum, Turkey

**Keywords:** aortic dissection, sex differences, tear-oriented repair, type A aortic dissection, surgical outcomes

## Introduction

I read with great interest the study by Matsumoto et al.,^1)^ which elegantly demonstrated that a tear-oriented surgical strategy yields comparable short- and long-term outcomes across sexes in acute Stanford type A aortic dissection (AAAD), despite markedly different dissection patterns and baseline profiles between male and female patients. The authors are to be commended for their rigorous single-center analysis and for applying multivariable logistic regression to confirm that female sex itself was not an independent predictor of in-hospital mortality (odds ratio 0.59, 95% confidence interval 0.15–2.09, p = 0.43). These findings carry meaningful clinical implications; however, we wish to offer several complementary perspectives that may further contextualize their results.

First, the sex-based anatomical divergence reported by Matsumoto et al., with female patients presenting more frequently with ascending aortic entry tears and limited distal extension, while male patients exhibited greater arch involvement and iliac propagation, aligns closely with the biological framework elaborated in our recent narrative review.^2)^ The distinct molecular structure of the female aorta, characterized by higher levels of matrix metalloproteinase-2 (MMP-2) and MMP-9 and an accelerated decline in tissue inhibitors of metalloproteinases (TIMP-1 and TIMP-2), constitutes an independent risk factor that shapes not only the propensity for dissection but also its anatomical pattern. Stiff, degenerated aortic walls in older postmenopausal women—the very cohort that predominated in the Matsumoto series, with a mean female age of 73.6 ± 9.7 years—may mechanically limit distal propagation, paradoxically producing a more surgically favorable dissection phenotype.^2)^ This biological underpinning helps explain why hemiarch replacement, with its lower procedural complexity and shorter circulatory arrest time, was sufficient in 89.4% of female patients without compromising durability.

Second, while the authors appropriately highlight the higher incidence of pericardial effusion in female patients (40.9% vs. 20.7%, p = 0.011), this finding warrants mechanistic consideration beyond rupture risk alone. Biomechanical data suggest that the female aortic wall exhibits reduced delamination resistance with advancing age, so that contained tamponade may reflect early transmural seepage rather than overt rupture.^1)^ The clinical implication is that pericardial effusion in elderly female AAAD patients should heighten urgency for intervention rather than prompt hesitation, a point the current data strongly support.

Third, the authors’ multivariable model included only age and sex as covariates, citing clinical relevance. We respectfully note that the Prognostic Nutritional Index and Geriatric Nutritional Risk Index were both significantly lower in female patients (p = 0.045 and <0.001, respectively) and that nutritional inflammatory frailty is an increasingly recognized predictor of adverse outcomes after aortic surgery.^3)^ Future analyses incorporating these indices, alongside preoperative D-dimer and inflammatory biomarker profiles, which carry independent prognostic value in AAAD,^2,4)^ may refine risk stratification beyond the binary sex variable and identify within-sex subgroups at elevated reoperative risk, particularly given the trend toward lower re-intervention-free survival in male patients (log-rank p = 0.099).

Finally, the Kaplan–Meier analyses demonstrated no significant difference in late re-intervention across sex and procedure subgroups; however, the follow-up attrition beyond 5 years acknowledged by the authors, combined with the relatively small female TAR subgroup (n = 4), limits the statistical power to detect late divergence. A multicenter registry with sex-stratified long-term surveillance is needed to definitively resolve whether tear-oriented limited repair in female patients remains durable beyond a decade, particularly in the context of residual false lumen patency, which, even at a lower rate in females (89.4% vs. 93.9%, p = 0.012), still represents an anatomical substrate for late aortic events.^5)^

In conclusion, the findings of Matsumoto et al. provide compelling evidence that individualized, tear-oriented surgical planning equalizes outcomes across sexes and should not be withheld from elderly female patients on account of age alone. Integrating sex-specific biomolecular and nutritional data into future prospective studies will be essential to fully harness the prognostic resolution that these anatomical observations promise.
